# Book Review: Surgery, the Ultimate Placebo

**DOI:** 10.3389/fsurg.2018.00038

**Published:** 2018-05-25

**Authors:** Jose D. Perezgonzalez

**Affiliations:** Business School, Massey University, Palmerston North, New Zealand

**Keywords:** surgery, science, evidence, scientific method, placebo, placebo effect.

What if many of the commonly performed surgeries were no better than placebo and surgeons performing them were no better than practitioners of alternative medicine?

That is the tantalizing message that Prof. Ian Harris has put forth in his latest book, a thought-provoking, well-researched and informative book addressed to a specialized audience within surgery and medicine but also of value to a general audience.

The book covers three topics throughout: the placebo effect (namely Chapters one, three, and eight), evidence-based practice (namely Chapters two and seven), and examples of contemporary and historical sham surgery (Chapters four, five and six). However, from the start (Introduction), the book already sets the basic contrast between placebo and evidence-based practice. The latter is identified with labels such as good science, evidence-based, experiment-based decision making, empirical, reality-centered, objective, and attending to specific therapeutic effects. The former is identified with labels such as bad science, tradition-based, assessment-based decision making, observational, perception-dependent, subjective, and biased by perceived therapeutic effects. The Introduction also contains the disclaimer that not all surgery is sham nor that surgeons intentionally perform sham surgery, and in so doing also sets the main focus of the book, which is on placebo surgery rather than on all surgery. The last chapter (Chapter nine) wraps up with recommendations for patients, doctors, researchers, funders, and society, in general.

What I find most compelling is that this is a book borne out of the professional experience of the author (Harris himself being an orthopedic surgeon) and reflects on the author’s own discovery of the evidence, or lack of thereof, behind his training and expertise. This makes the book a manifesto for science in medicine, in general, and for the use of the scientific method in surgery, in particular.

Indeed, because Harris is a trained surgeon, he knows first-hand the ins and outs of how surgeons are trained— “Medical (and particularly surgical) training. . . is too much of an apprenticeship” (p. 198); and “surgeons tend to dwell on the technical aspects of surgery” (p. 31)—, how they think—“There is a saying in surgery: any surgeon can operate; a good surgeon knows *when* to operate, but the best surgeons know when *not* to operate (p. 186); “largely, surgeons believe that they are doing the right thing” (p. 2); while “the proponents of any particular operation usually have a scientific explanation to justify the treatment” (p. 22)—and of the multiple opportunities and limitations that influence a surgeon’s ultimate decision whether to operate or not—“To a man with a hammer, everything looks like a nail. Surgeons operate – it is what they do” (p. 256); plus the plethora other reasons behind the persistence of ineffective surgery (Chapter 7).

Above training, thinking, and decision-making mean for Harris that surgeons are very well-trained and capable technicians but not necessarily versed in the science that supports their trade. For the technical aspects of surgery “may be relevant, but they are not necessarily aligned with a measurable clinical benefit for the patient” (p. 31); surgeons thinking (or assuming) they are doing the right thing does not imply they are “aware of the strength (or weakness) of the supporting evidence” (p. 2); and “a scientific plausible explanation is no guarantee that the treatment works” (p. 22). Thus, it is here where Harris own inroad into the scientific method, the scientific debunking of non-scientific beliefs, and into evidence-based medicine provides value to surgery.

Ultimately, science and the practice of medicine are two separate worlds with different ethical standards. “Science takes a global, societal perspective in which the risk to the individual is balanced against the potential good to society. The practice of medicine tends to concentrate on the individual, avoiding harm or risk wherever possible” (p. 81). That is, while science researches populations, anonymizing the individual in favor of group statistics, surgeons necessarily decide at the individual level, where idiosyncrasies occur. And although Harris’s book shows awareness of both worlds, it far too readily dismisses the latter in favor of the former. Furthermore, the science that Harris portrays is one based on frequentist statistics, on ascertaining the probability of surgery effectiveness when assuming that it is not going to be different than placebo. But surgical practice implies a Bayesian mindset, of ascertaining the probability of making a difference (e.g., of a positive outcome) if performing surgery (successfully). Ironically, even the science that Harris proposes is based on the simpler tests of statistical significance, where only a difference can be tested but nothing is warranted otherwise (i.e., lack of evidence of surgery effectiveness is not, in itself, evidence of its lack of effectiveness). Despite those shortcomings, however, Harris makes a good attempt to bridge both worlds by book’s end, making it a worthwhile reference in a surgeon’s library.

## Author Contributions

The author confirms being the sole contributor of this work and has approved it for publication.

## Conflict of Interest Statement

The author declares that the research was conducted in the absence of any commercial or financial relationships that could be construed as a potential conflict of interest.

